# Early continuous veno-venous haemofiltration in the management of severe acute pancreatitis complicated with intra-abdominal hypertension: retrospective review of 10 years' experience

**DOI:** 10.1186/2110-5820-2-S1-S21

**Published:** 2012-12-20

**Authors:** Guntars Pupelis, Haralds Plaudis, Kaspars Zeiza, Nadezda Drozdova, Maksims Mukans, Ita Kazaka

**Affiliations:** 1 Department of General and Emergency Surgery, Riga East Clinical University Hospital 'Gailezers', Hipokrata str. 2, Riga, 1038, Latvia

## Abstract

**Background:**

Conservative treatment of patients with severe acute pancreatitis (SAP) may be associated with development of intra-abdominal hypertension (IAH), deterioration of visceral perfusion and increased risk of multiple organ dysfunction. Fluid balance is essential for maintenance of adequate organ perfusion and control of the third space. Timely application of continuous veno-venous haemofiltration (CVVH) may help in balancing fluid replacement and removal of cytokines from the blood and tissue compartments. The aim of the present study was to determine whether CVVH can be recommended as a constituent of conservative treatment in patients with SAP who suffer IAH.

**Methods:**

A retrospective analysis of 10 years' experience with low-flow CVVH application in patients with SAP who develop IAH was. In all patients, measurement of the intra-abdominal pressure (IAP) was done indirectly through the urinary bladder. Sequential organ failure assessment (SOFA) score was calculated for severity assessment, and necrotizing forms were verified by contrast-enhanced computed tomography. Dynamics of IAP were analysed in parallel with signs of systemic inflammation, dynamics of C-reactive protein and cumulative fluid balance. All variables, complication rate and outcomes were analysed in the whole group and in patients with IAH (CVVH and no-CVVH groups).

**Results:**

From the total of 130 patients, 75 were treated with application of CVVH and 55 without CVVH. Late hospitalization was associated with application of CVVH. Infection was observed in 28.5% of cases regardless of the type of treatment received, with a similar necessity for surgical intervention. IAH was observed in 68.5% of patients, and they had significantly higher SOFA scores compared to patients with normal IAP. CVVH treatment resulted in negative cumulative fluid balance starting from day 5 in patients with IAH, whereas without this treatment, fluid balance remained increasingly positive after a week. Finally, application of CVVH resulted in a lower infection rate and shorter hospital stay, 26.7% vs. 37.9%, and a median of 32 (interquartile range (IQR) = 60 to 12) days vs. 24 (IQR = 34 to 4) days, *p *= 0.05, comparing CVVH vs. no-CVVH group. Mortality rate reached 11.7% in the CVVH group and 13.8% in the no-CVVH group.

**Conclusions:**

Early application of CVVH facilitates negative fluid balance and reduction of IAH in patients with SAP; it is not associated with increased infection or mortality rate and may reduce hospital stay.

## Background

Severe acute pancreatitis (SAP) manifests itself with local inflammation, which involves the pancreas and surrounding tissue, and systemic inflammation with characteristic systemic increase of vascular permeability. Exudation with inflammatory fluid accumulations in intra-abdominal, retroperitoneal and pleural cavities is characteristic of SAP (grade E according to the Balthazar computed tomography severity index) [1]. Retroperitoneal and intraperitoneal distribution of inflammatory fluid and visceral oedema may cause elevation of intra-abdominal pressure (IAP) with resultant intra-abdominal hypertension (IAH) [2]. Prolonged IAH affects visceral perfusion and organ function, leading to the development of multiple organ dysfunction (MODS) and impairment of bowel barrier function and thus increasing the risk of bacterial translocation and septic complications [3]. In the early phase, severity of the clinical course depends on the magnitude of systemic inflammation and anti-inflammatory capacity of the immune response [4]. Disbalanced inflammatory response facilitates invasion of infection, leading to poor prognosis with 30% to 50% mortality [5]. Fast reduction of inflammatory fluid accumulation and decrease of visceral oedema improve early treatment prognosis considerably, while late prognosis generally depends on the presence of infection [6]. Management of IAH and the abdominal compartment syndrome (ACS) includes conservative measures; however, when they fail, surgical treatment is the only remaining option. In the line of conservative treatment modalities, continuous veno-venous haemofiltration (CVVH) is positioned mostly as an option for treatment of renal dysfunction with relatively low C grade of evidence [7]. It is proved that application of CVVH facilitates removal of cytokines and biologically active substances from the blood and also from the extravascular compartment, reducing fluid sequestration in the third space [8]. This retrospective study was aimed to summarize our 10 years of experience in the clinical application of CVVH as well as to determine the incidence of IAH in SAP patients, assess the impact of CVVH on IAP and evaluate available data for prediction of outcomes in SAP patients who suffer from IAH.

## Methods

### Patient selection

Medical histories of SAP patients who were admitted to Riga East Clinical University Hospital 'Gailezers' during the period from January 2000 to June 2010 were analysed retrospectively. SAP was diagnosed according to the Atlanta criteria based on the clinical course of the disease, a threefold increase in lipase activity in plasma and one of the following criteria: systemic inflammation and/or signs of organ dysfunction, and acute physiology and chronic health evaluation (APACHE) II score >8 [9]. Severity assessment was done by calculation of sequential organ failure assessment (SOFA) score for recognition of organ dysfunction [10]. Necrotizing forms were verified by contrast-enhanced computed tomography (CECT) scan and elevated C-reactive protein (CRP) level above 250 mg/L.

### Abdominal pressure measurement

IAP was measured and recorded at least twice daily indirectly through the urinary bladder after instillation of a 50-mL sterile saline solution; we used the symphysis as the reference point before the latest recommendations of the World Society of the Abdominal Compartment Syndrome (WSACS) were published in November 2006 [11]. Since April 2007, we followed WSACS guidelines which recommend supine position of the patient, instillation of a 20-mL sterile saline solution and the linea axillaris media and crista iliaca cross point as the zero point. The results were expressed in millimetres of mercury (mmHg). Measurement techniques did not demonstrate significant differences in measurement results before and after April 2007.

### Definitions

IAH was defined by a sustained or repeated pathological elevation in IAP ≥ 12 mmHg. ACS was diagnosed when sustained increase of the IAP > 20 mmHg and one new organ dysfunction were detected [11].

### Treatment protocol

All patients were treated according to standardized treatment protocol, and the only difference was whether CVVH was applied during the treatment course. The main indications for CVVH were the following: evidence of increased exudation due to progression of systemic inflammation, and formation of multiple fluid collections accompanied by deterioration of respiratory and kidney functions, despite the 24- to 48-h intensive treatment provided according to the approved treatment protocol of SAP. Development of MODS and sustained increase of IAP ≥12 mmHg in these patients was a strong indicator for commencement of CVVH. The procedure was performed using Diapact CRRT B.Braun Co (Melsungen, Germany) or Fresenius Medical Care Multifiltrate machines (Bad Homburg, Germany). Synthetic high-volume membrane filters with a surface area of 1.5 to 2.2 m^2 ^were used and changed every 24 h or when filter blocking occurred. Vascular access was obtained by a double- or triple-lumen catheter using the femoral or jugular vein. Anticoagulation was provided with non-fractioned heparin, adjusting the dosage according to the value of activated partial thromboplastin time in the plasma. The procedure was performed with a low dose of heparin or without it when possible. The substitution fluid infusion rate was 1,000 to 1,460 mL/h in a pre-diluted or post-diluted manner, comprising 24 to 35 L of the total substitute in 24 h. The blood flow rate was 50 to 200 mL/min. Ultrafiltration rate was adjusted according to the diuresis and fluid balance.

The conservative treatment strategy was identical in both groups and included isovolemic haemodilution with early and adequate colloid infusion, oxygen supply, stimulation of kidney function, other organ support and intravenous antibacterial prophylaxis with fluoroquinolones 400 mg twice daily and metronidazole 500 mg three times a day or imipenem/cilastatin monotherapy 500/500 mg four times daily. On routine basis, within 48 to 72 h from admission, we commenced low-volume enteral nutrition with iso-osmolar, whole protein low-fat enteral feeding formulas, which helped in the stimulation of the gut and recovery of the gut transit function. Indications for early surgical intervention were obscure diagnosis or failure to control sustained increase of the IAP with conservative treatment. Late surgical interventions were done in cases when infection of pancreatic/peripancreatic necrosis complicated the clinical course.

### Follow-up and outcome prediction

All patients were assessed regarding the severity and clinical course of the disease. Complications and outcomes were analysed comparing treatment results in patients who underwent CVVH and those who did not undergo CVVH. For patients with IAH, a separate analysis of severity, complication rate and outcomes was performed, grouping patients with IAH in CVVH and no-CVVH groups.

Measurements of IAP were analysed in parallel with the assessment of the degree of organ dysfunction using SOFA score, CRP and activity of serum lipase.

Our major endpoint was hospital mortality. Secondary endpoints were incidence of septic complications, length of stay in intensive care unit (ICU) and overall hospital stay.

### Statistics

Data are presented as mean with standard deviation when normally distributed and as median with interquartile range (IQR) in case of non-normal distribution. Analysis was performed with the Student's *t *test, Mann-Whitney test or the likelihood ratio test whenever appropriate. Categorical data were compared and assessed by Chi-square test, and significance was verified by Fisher's exact test. Univariate analysis was performed separately on patients with IAH, comparing patients without IAH, as well as between the CVVH group and the no-CVVH group. Additionally univariate analysis was performed to compare data in survivors vs. non-survivors on the whole patient group - to see whether IAP is a predictor for outcome.

Stepwise multiple logistic regression analysis was used to find an independent predictor of mortality and to verify its significance. Multiple logistic regression analysis was done to identify predictive factors for IAH. To ascertain the cut-off values and verify the significance of the predicting value of IAP and abdominal perfusion pressure (APP), receiver operating characteristic (ROC) curve analysis was performed with calculation of the area under the ROC curve. Statistical analysis was done using SPSS version 17.0 (IBM Corporation, Chicago, IL, USA).

## Results

### Demographics, incidence of IAH, organ failure and complication rate in whole group

**Figure 1:**
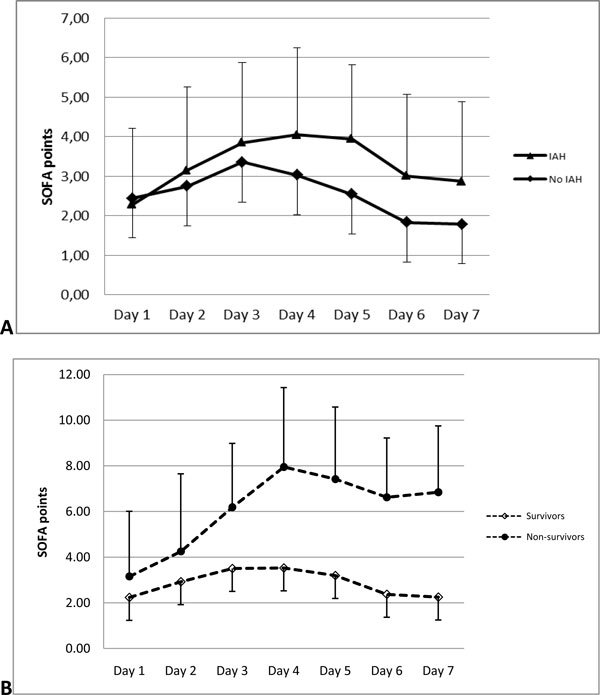
**Dynamics of SOFA score**. (A) **Patients with IAH and normal IAP in whole group**. Significant difference on day 4 (*p *= 0.04), day 5 (*p *= 0.01) and day 6 (*p *= 0.04). (B) **Survivors and non-survivors in whole group**. Significant difference on day 3 (*p *= 0.004), day 4 (*p *= 0.001), day 5 (*p *= 0.0006) and day 6 (*p *= 0.0008).

The mean age of patients was 47.6 years, male patients dominated, and IAH was more often found in males. The overall incidence of IAH was 68.5%; however, incidence of initial organ failure was the same among patients with normal IAP compared to patients who developed IAH. It became evident that patients with IAH were hospitalized significantly later and underwent CVVH more often (Table 1). The SOFA score was significantly increased in patients with IAH on day 4 after admission and commencement of therapy. This elevation was observed for the next 2 consecutive days, and then SOFA score decreased in both groups (Figure 1). In total, 75 patients underwent CVVH and 55 patients were treated without application of CVVH. The overall incidence of infection was 28.5% regardless of the type of treatment with similar necessity for surgical intervention in patients with normal IAP and those who developed IAH (Table 1).

**Table 1:** Demographics, incidence of IAH, organ failure and complication rate in whole group

	Total group *N *= 130	IAH *N *= 89	no IAH *N *= 41	*p *value
Age, years (range)	47.6 ± 15.4 (19 to 84)	47.1 ± 14.6 (21 to 84)	48.6 ± 17.0 (19 to 81)	NS
Male, *n *(%)	95 (73.1%)	70 (78.7%)	25 (61.0%)	0.035
Time from the first symptoms to hospitalization, median hours (IQR)	20 (48 to 4)	24 (48 to 5.8)	13 (22.5 to 0.5)	0.001
APACHE II at admission, points	7.6 ± 4.5	7.8 ± 4.7	7.1 ± 4.0	NS
IAP on admission, mmHg	13 ± 3.8	14.9 ± 3.8	10.5 ± 1.7	0.001
SOFA on admission, points	2.3 ± 2.1	2.3 ± 2.1	2.4 ± 1.9	NS
Necrotizing SAP, *n *(%)	89 (68.5%)	65 (73.0%)	24 (58.5%)	NS
MODS, *n *(%)	122 (93.9%)	84 (94.4%)	38 (92.7%)	NS
Underwent CVVH, *n *(%)	75 (57.7%)	60 (67.4%)	15 (36.6%)	0.001
Pancreatic/peripancreatic infection, *n *(%)	37 (28.5%)	27 (30.3%)	10 (24.4%)	NS
Surgical intervention, *n *(%)	36 (27.7%)	26 (29.2%)	10 (24.4%)	NS

### Influence of CVVH treatment on whole group

Just like in the whole group, males dominated in the group of patients who underwent CVVH; they were admitted to the hospital significantly later in the course of the disease and had significantly higher IAP measurements on admission, with more frequent development of necrotizing forms compared to patients who did not undergo CVVH. Commencement of the procedure was started within the first 48 h after admission with a median duration of 72 (IQR = 119 to 44.8) h in 78% of patients. Development of metabolic acidosis and a higher rate of pulmonary complications were significant among patients who underwent CVVH; however, infection rate and the need for surgical treatment were the same compared to patients treated without CVVH (Table 2).

**Table 2:** Impact of CVVH treatment on whole group

		Treatment		
		CVVH *n *= 75	no CVVH *n *= 55	*p *value
Demographics	Age, years	44.7 ± 13.9	51.5 ± 16.6	NS
	Male, *n *(%)	65 (86.7%)	31 (56.4%)	
	Time from the first symptoms to hospitalization, median hours (IQR)	16 (26.8 to 3.8)	19.5 (48 to 11.5)	NS
	APACHE II at admission, points	7.8 ± 4.1	7.5 ± 4.8	NS
	IAP on admission, mmHg	14.2 ± 4.0	11.8 ± 3.0	0.048
	SOFA on admission, points	2.4 ± 2.2	2.2 ± 1.9	NS
	Necrotizing SAP, *n *(%)	62 (82.7%)	28 (50.9%)	<0.001
	MODS, *n *(%)	70 (93.3%)	53 (96.3%)	NS
Organ dysfunctions	Renal dysfunction, *n *(%)	32 (42.7%)	15 (27.3%)	NS
	Pulmonary dysfunction, *n *(%)	12 (16.0%)	5 (9.1%)	NS
	Liver dysfunction, *n *(%)	12 (16.0%)	6 (10.9%)	NS
	Cardiovascular, *n *(%)	6 (8%)	3 (5.5%)	NS
	Hematologic, *n *(%)	5 (6.7%)	4 (7.2%)	NS
	Neurologic, *n *(%)	15 (20%)	6 (10.9%)	NS
Complications	Metabolic acidosis, *n *(%)	24 (32%)	7 (12.7%)	0.013
	Pleural effusion, *n *(%)	47 (62.7%)	14 (25.5%)	<0.001
	Pneumonia, *n *(%)	31 (41.3%)	5 (9.1%)	<0.001
	Atelectasis, *n *(%)	11 (14.7%)	2 (3.6%)	0.043
	Pancreatic/peripancreatic infection, *n *(%)	21 (28%)	16 (29.1%)	NS
	Surgical intervention, *n *(%)	21 (28%)	15 (27.3%)	NS

### Management of patients with IAH

**Figure 2:**
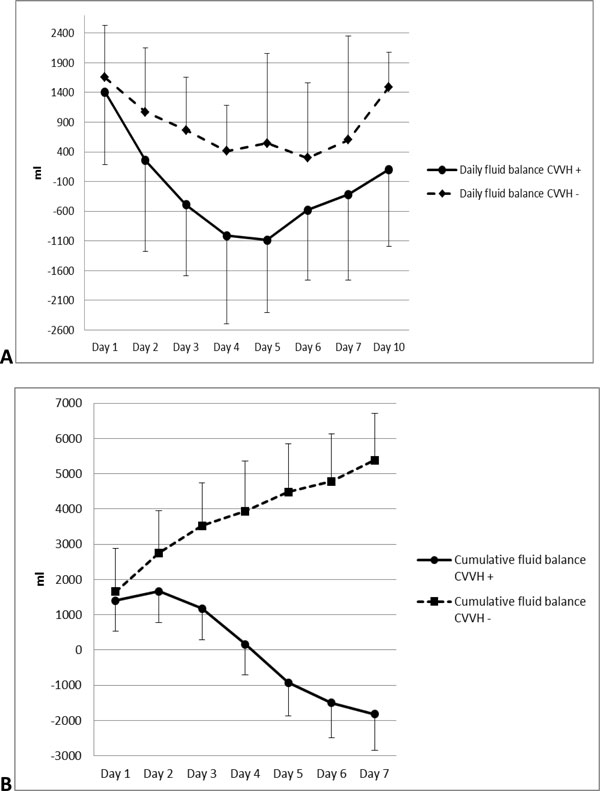
**Dynamics of daily and cumulative fluid balance**. **(A) **Daily fluid balance. Significant difference on day 3 (*p *= 0.02), day 4 (*p *= 0.007) and day 5 (*p *= 0.007). **(B) **Cumulative fluid balance. Significant difference on day 2 (*p *= 0.05), day 3 (*p *= 0.001), day 4 (*p *= 0.0008), day 5 (*p *= 0.0008), day 6 (*p *= 0.0006) and day 7 (*p *= 0.0006).

For further assessment, patients with IAH were divided in CVVH group and no-CVVH group. MODS complicated the clinical course in both groups equally. In the beginning of the treatment, 65% of patients from CVVH group had signs of renal dysfunction. The mean creatinine value before the start of CVVH was 162.7 ± 124.8 μmol/L; 26 patients (43.3%) in this group had oliguria, while only 6 (20.7%) oliguric patients were treated without CVVH (*p *= not significant (NS)). Radiologic evidence of pleural effusion and pneumonia was typical for patients with multiple inflammatory fluid collections; most of them underwent CVVH. During the first week of treatment, development of pulmonary complications and metabolic acidosis were observed significantly more often in the CVVH group, correlating with the significantly higher rate of necrotizing forms. ACS developed approximately in one third of patients, without significant differences between the groups (Table 3). Vasopressor support was needed for eight patients (13.3%) in the CVVH group and for two patients (6.9%) in the no-CVVH group (*p *= NS). Ventilatory support during the conservative treatment period was used in 15 patients (25%) from the CVVH group and in 2 (6.9%) patients from the no-CVVH group (*p *= 0.048). Mean values of cumulative fluid balance in CVVH group were close to 0 on day 4 after commencement of conservative treatment and significantly different compared to no-CVVH group. Starting from day 5, it became negative (Figure 2).

**Table 3:** Application of CVVH in patients with IAH

		CVVH *N *= 60	No CVVH *N *= 29	*p*
Demographics	Age, years	45.1 ± 13.4	51.3 ± 16.4	NS
	Male, *n *(%)	52 (86.7%)	18 (62.1%)	
	Time from the first symptom to hospitalization, median hours (IQR)	32 (60 to 12)	24 (34 to 4)	NS
	APACHE II at admission, points	7.9 ± 5.1	7.2 ± 4.2	NS
	IAP on admission, mmHg	15.4 ± 3.7	14.5 ± 3.7	NS
	SOFA on admission, points	2.4 ± 2.2	2.0 ± 1.9	NS
	Necrotizing SAP, *n *(%)	49 (81.7%)	16 (55.2%)	0.01
	MODS, *n *(%)	58 (96.7%)	26% (89.7)	NS
	ACS, *n *(%)	23 (38.3%)	10 (34.5%)	NS
Organ dysfunctions	Renal, *n *(%)	27 (45%)	8 (27.6%)	NS
	Pulmonary, *n *(%)	11 (18.3%)	3 (10.3%)	NS
	Liver, *n *(%)	6 (10%)	3 (10.3%)	NS
	Cardiovascular, *n *(%)	5 (8.3%)	2 (6.9%)	NS
	Hematologic, *n *(%)	4 (6.7%)	3 (10.3%)	NS
	Neurologic, *n *(%)	15 (25%)	4 (13.8%)	NS
Complications	Metabolic acidosis, *n *(%)	15 (25%)	2 (6.9%)	0.05
	Pleural effusion, *n *(%)	40 (66.7%)	11 (37.9%)	0.01
	Pneumonia, *n *(%)	28 (46.7%)	4 (13.8%)	0.002
	Atelectasis, *n *(%)	8 (13.3%)	2 (6.9%)	NS
	Pancreatic/peripancreatic infection, *n *(%)	16 (26.7%)	11 (37.9%)	NS
	Surgical intervention, *n *(%)	16 (26.7%)	10 (34.5%)	NS

**Figure 3:**
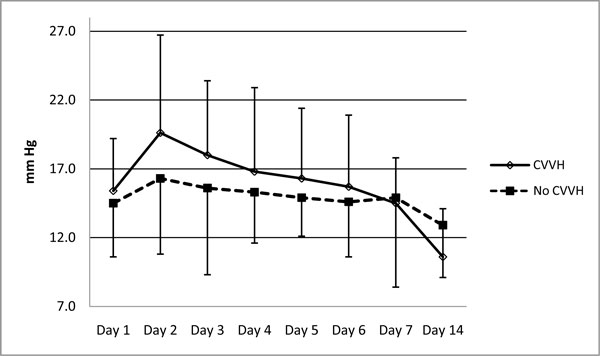
**Dynamics of IAP with and without CVVH treatment**. **Significant difference on day 2 (***p ***= 0.02)**.

**Figure 4:**
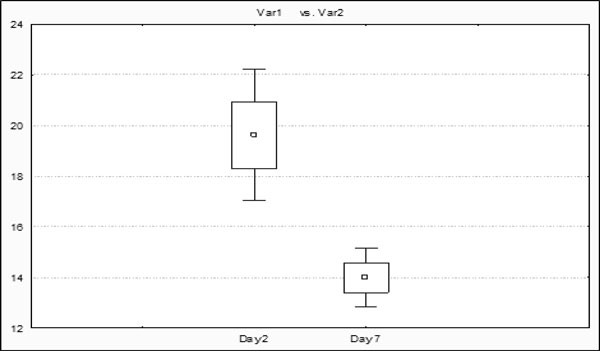
**Drop of IAP in CVVH group**. Significant drop of IAP was seen in CVVH group comparing day 2 and day 7.

CVVH treatment influenced the dynamics of IAP positively. After commencement of conservative therapy, a similar elevation of the IAP was observed in both groups on day 1. On day 2, IAP reached 19.6 ± 7.1 mmHg in patients who later underwent CVVH vs. 16.3 ± 5.5 mmHg in the no-CVVH group (*p *= 0.05). Application of CVVH resulted in faster decrease of IAP during the first phase of the disease when IAP reached 10.6 ± 3.9 mmHg within 2 weeks, while the mean IAP in patients treated without CVVH was still elevated at 12.9 ± 4.1 mmHg (Figures 3 and 4).

### Systemic inflammation and infection rate

**Figure 5:**
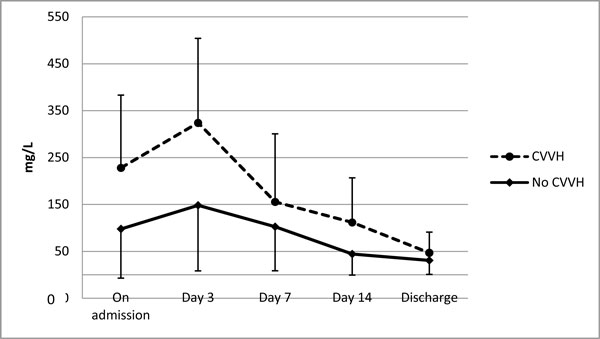
**Dynamics of CRP in patients with IAH secondary to mode of treatment**. **Significant difference on day 3 (***p ***= 0.05)**.

On admission, CRP, the marker of systemic inflammation, was significantly higher in the CVVH group with 227.9 ± 128.8 mg/L compared to 97.7 ± 62.8 mg/L in the no-CVVH group (*p *= 0.02). The difference increased on the third day after commencement of the therapy reaching 324.5 ± 179.3 mg/L vs. 153.7 ± 91.6 mg/L (*p *= 0.044), respectively, and normalized in both groups during a 2-week period. CVVH application time correlated with a faster decrease of CRP (Figure 5). Decrease of lipase activity in the serum was rapid in both groups, dropping within the first 48 h from 2,493.2 ± 1247.6 U/L to 589.6 ± 346.4 U/L and from 2,074 ± 971.3 U/L to 426.4 ± 297.8 U/L similarly in the CVVH group and the no-CVVH group.

The conservative therapy with application of CVVH resulted in a lower infection rate, and consequently, surgical treatment was necessary in only 26.7% of patients from this group compared to 34.5% of patients in the no-CVVH group (Table 3).

### Main outcomes

**Figure 6:**
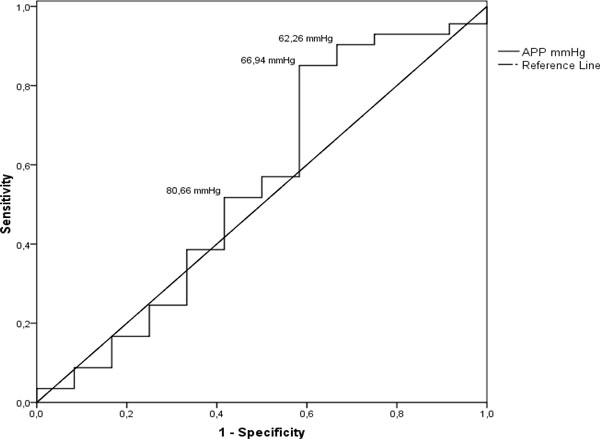
**ROC curve**. Mortality predictive values of APP. The curve describes the association between sensitivity and specificity at different thresholds of APP in predicting mortality. Cut-off values shown in the figure are 80.66 mmHg (true positive rate 57%, false positive rate 50%), 66.94 mmHg (true positive rate 85.1%, false positive rate 41.7%) and 62.26 mmHg (true positive rate 90.4%, false positive rate 33.3%). ROC curves that approach the upper leftmost corner represent highly accurate studies - in this case, the area under the curve (AUC) is 0.548. Accuracy of APP in predicting mortality has failed; also this ROC curve analysis is not significant - *p *value 0.583.

**Figure 7:**
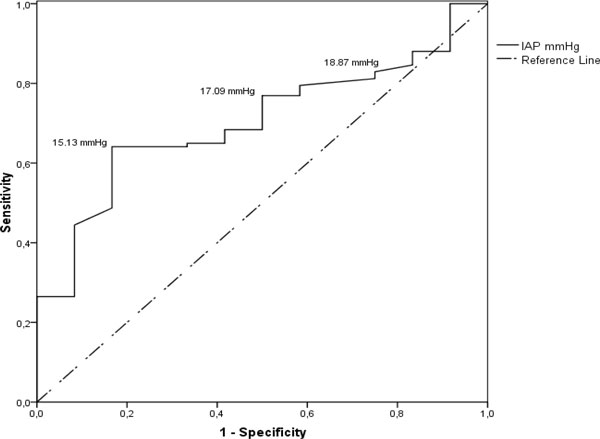
**ROC curve**. Mortality predictive values of IAP. The curve describes the association between sensitivity and specificity at different thresholds of IAP in predicting mortality. Cut-off values shown in the figure are 15.13 mmHg (true positive rate 65%, false positive rate 68.3%), 17.09 mmHg (true positive rate 76.9%, false positive rate 50%) and 18.87 mmHg (true positive rate 82.9%, false positive rate 25%). ROC curves that approach the upper leftmost corner represent highly accurate studies - in this case AUC is 0.703. Accuracy of IAP in predicting mortality is fair, and ROC curve analyses is statistically significant - *p *value 0.021.

To assess the main outcomes, we took the following steps. First of all, we assessed main outcomes among all patients and did not discover significant differences in regard to CVVH application. However, the overall hospital stay was significantly longer in patients with IAH, while ICU stay was not different compared to patients with normal IAP. Analysis according to the likelihood test revealed significant difference in mortality. Patients with IAH had a higher death rate compared to patients with normal IAP, but Fisher's exact test did not reveal the difference. We then assessed outcomes only in patients with IAH and discovered that application of CVVH resulted in a shorter hospital stay compared to patients with IAH who were treated without CVVH (Table 4). Application of univariate analysis revealed that non-survivors were operated more often (Table 5). Logistic regression analysis found that IAH is an independent predictor of mortality (*p *= 0.043), while renal dysfunction (*p *= 0.011) and pleural effusion (*p* < 0.001) are independent predictors of IAH. Finally, we performed ROC analysis to look for thresholds of IAP and APP that are predictive for mortality. Analysis showed that APP is not a suitable predictor of mortality; however, IAP is a significant predictor of mortality with high sensitivity at the level of 17.09 mm Hg (Figures 6 and 7).

**Table 4:** Main outcomes

		ICU stay, median days (IQR)	Hospital stay, median days (IQR)	Mortality, *n *(%)
All patients *n *= 130		9 (14 to 5.5)	20 (31 to 15)	12 (9.2%)
Influence of CVVH on outcome in whole group	CVVH *n *= 75	12 (11 to 5)	20 (32 to 15)	8 (10.7%)
	no CVVH *n *= 55	7 (16.5 to 6)	19 (27 to 15)	4 (7.3%)
	*p *value	NS	NS	NS
Influence of IAH on outcome in whole group	IAH *n *= 89	10 (16 to 6)	32 (16 to 6)	11 (12.4%)
	Normal IAP *n *= 41	6 (9 to 4)	16 (23.5 to 13)	1 (2.4%)
	*p *value	NS	0.05	0.044^a^
Influence of CVVH on outcome in patients with IAH	CVVH *N *= 60	9 (16 to 6)	32 (60 to 12)	7 (11.7%)
	No CVVH *N *= 29	10 (16 to 7.5)	24 (34 to 4)	4 (13.8%)
	*p *value	NS	0.05	NS

^a^Fisher's NS, likelihood significant.

**Table 5:** Univariate analysis

	Survivors *N *= 118	Non-survivors *N *= 12	*p *value
MODS, number of cases (%)	110 (93.2%)	11 (91.7%)	NS
Necrotizing SAP, number of cases (%)	80 (67.8%)	10 (83.3%)	NS
Surgery, number of cases (%)	42 (35.6%)	9 (75.0%)	0.01
ICU stay, median days (IQR)	8 (14 to 6)	11.5 (19.3 to 5)	NS
Hospital stay, median days (IQR)	20 (31 to 15)	24 (33.5 to 16.3)	NS
IAP on admission, mmHg	14.8 ± 4.2	16.4 ± 1.5	NS

## Discussion

The aim of this retrospective study was to summarize our experience in the clinical application of CVVH in SAP patients who develop IAH. Our data demonstrate that male patients in their late 40s who were hospitalised after a 24-h period from the appearance of the first symptoms frequently had increased IAP on admission. Partially, it can be explained by the high number of alcohol abusers among these patients, with their often careless attitude to health. The overall incidence of IAH in our study group was 68.5%, demonstrating clinical significance of this pathophysiologic phenomenon. However, these results show indirectly that, in majority of the cases, the increase of the IAP was observed after commencement of the conservative treatment that included balanced fluid replacement therapy and organ support for improvement of tissue perfusion [7]. Reaching the optimal fluid balance is quite a challenging task, and failure of the initial treatment can lead to development of IAH, progressive deterioration of tissue perfusion and organ dysfunction [12]. Current knowledge about the treatment of IAH and ACS is somewhat controversial; while CVVH is mentioned as a treatment modality, it is by far not the leading one [13]. Preventive treatment is the most effective approach; however, a number of publications have given emphasis to the treatment of ACS, making analysis of how to prevent IAH less prominent.

Our strategy was based on evaluation of the 24-h conservative treatment response. Negative dynamics of systemic inflammation, increase of the IAP and deterioration of the organ function were critical signs for reconsideration of our therapeutic strategy and application of CVVH. More than half of our patients underwent CVVH within the first 48 h from admission. Patients with IAH were the first candidates for the procedure because 65% of them had signs of renal failure with significant incidence of metabolic acidosis. Majority of them were hospitalized late and had necrotizing SAP. During the early phase of the disease, they developed pulmonary complications more often. Application of CVVH resulted in effective decrease of IAP, and it correlated with the achievement of negative fluid balance. Our study was not aimed at analysis of the particular mode of CVVH due to the fact that low substitution rate was recommended by renal replacement therapy specialists; however, application of low-flow haemofiltration in patients with IAH is mentioned as an optional strategy leading to promising results also by other authors [14]. The mode of haemofiltration is a matter of debate since not only the mechanical removal of cytokines from the blood and reduction of tissue cytokine effects could be important. Several authors argue against low-flow CVVH, instead reporting promising experience with high-flow haemofiltration [15,16]. However, the reported series are small and lack adequate severity assessment. Due to the retrospective design of the study, evaluation of severity, systemic inflammatory response and organ failure was based on assessment of routine clinical data, and this certainly limits precise interpretation of the results. Nevertheless it is evident that CVVH was started in the risk group even before the peak elevation of the CRP, giving some ground for speculation that it could work in a preventive manner. Critical aspects of the procedure may be invasiveness and increased infection risk. Analysis in all patients and in patients with IAH did not demonstrate increased complication risk associated with the procedure. Moreover, application of CVVH resulted in a lower infection rate in patients with IAH and consequently in fewer surgical interventions. This can be partially explained by faster reduction of tissue compartments and exudate collections, improving local tissue resistance to bacterial invasion or improving gut barrier function [17]. Although reduction of the infection rate by prophylactic antibacterial treatment is not proved, it was a constituent of our treatment protocol and complies with recent recommendations demonstrating benefits from broad-spectrum antibacterial prophylaxis in cases of necrotizing pancreatitis proven by CECT [18,19].

We provided outcome analysis in several aspects. Overall ICU stay, hospital stay and mortality rate were similar in patients who underwent CVVH and who were treated without CVVH, corresponding to internationally available data favouring multidisciplinary approach and postponed surgery [20]. IAH was associated with increased mortality when we performed grouping of patients according to the degree of IAP. Application of CVVH in patients with IAH resulted in reduced hospital stay but did not significantly change ICU stay or mortality. Univariate analysis revealed that mortality is associated with surgical interventions and longer ICU stay. Finally, the logistic regression analysis revealed that renal dysfunction and pleural effusion are independent predictors of IAH, but IAH is an independent predictor of mortality. The predicting value of IAP according to ROC analysis was 14.8 mmHg. It means that elevation of the IAP to this level could be a trigger for a revision of the treatment strategy. Our results demonstrate that application of CVVH can work preventively through achievement of negative cumulative fluid balance and does not increase complication rate, infection risk or mortality.

Our retrospective study has certain limitations due to poor demographic data and relatively long time period chosen for data analysis. The SOFA score was calculated routinely for severity assessment, while APACHE II score was calculated only on admission. However, during the last 10 years, we have applied standardized conservative treatment protocol, which allows us to suggest that the overall treatment success can be evaluated with certain precision. Changes in the IAP measurement technique according to our data did not significantly influence values of IAP measurements. We did not analyse a few rare occasions when paracentesis or other nonsurgical methods were used for control of the IAP because, if these methods were successful, patients did not undergo CVVH. Summarizing our 10 years of experience in the clinical application of CVVH, we would recommend it as a rational constituent of the conservative treatment protocol in patients with SAP who suffer from sustained increase of the IAP.

## Conclusions

Early application of CVVH facilitates negative fluid balance and reduction of IAH in patients with severe acute pancreatitis; it is not associated with increased infection or mortality rate and may reduce hospital stay.

## Abbreviations

ACS: abdominal compartment syndrome; APACHE: acute physiology and chronic health evaluation; APP: abdominal perfusion pressure; CECT: contrast-enhanced computed tomography; CRP: C-reactive protein; CVVH: continuous veno-venous haemofiltration; IAH: intra-abdominal hypertension; IAP: intra-abdominal pressure; ICU: intensive care unit; IQR: interquartile range; MODS: multiple organ dysfunction syndrome; SAP: severe acute pancreatitis; SOFA: sequential organ failure assessment; ROC: receiver operating characteristic; WSACS: World Society of the Abdominal Compartment Syndrome.

## Competing interests

Authors declare that they have no competing interests.

## Authors' contributions

GP contributed to the conceiving of the study, interpretation of the results, drafting and revision of the manuscript and approval of the manuscript in its final form. HP and KZ contributed to the treatment of the patients and acquisition and management of the data. MM contributed to the performance of statistical analysis and acquisition of the data. ND and IK contributed to the acquisition of the data and provision of data input in the electronic data base. All authors read and approved the final manuscript.
